# Atypical working hours are associated with tobacco, cannabis and alcohol use: longitudinal analyses from the CONSTANCES cohort

**DOI:** 10.1186/s12889-022-14246-x

**Published:** 2022-09-29

**Authors:** Nadine Hamieh, Guillaume Airagnes, Alexis Descatha, Marcel Goldberg, Frédéric Limosin, Yves Roquelaure, Cédric Lemogne, Marie Zins, Joane Matta

**Affiliations:** 1 INSERM, Population-based Epidemiological Cohorts Unit, UMS 011, Villejuif, France; 2 Université Paris Cité, Faculty of Health, School of Medicine, Université Paris Cité, F-75006 Paris, France; 3 AP-HP.Centre-Université de Paris, DMU Psychiatrie et Addictologie, Centre Ambulatoire d’Addictologie, Hôpital européen Georges-Pompidou, F-75015 Paris, France; 4 Poison Control Center, Academic Hospital CHU Angers, F-49000 Angers, France; 5 Univ Angers, Centre Hospitalier Universitaire CHU Angers, Université de Rennes, INSERM, École des hautes études en santé publique, Institut de recherche en santé, environnement et travail Irset UMR_S 1085, F-49000 Angers, France; 6 Department of Occupational Medicine, Epidemiology and Prevention, Donald and Barbara Zucker School of Medicine, Hofstra/Northwell, Hempstead, USA; 7 Université Paris Cité, INSERM U1266, Institut de Psychiatrie et Neuroscience de Paris, F-75014 Paris, France; 8 AP-HP.Centre-Université de Paris, DMU Psychiatrie et Addictologie, Service de psychiatrie et d’addictologie de l’adulte et du sujet âgé, Hôpital Corentin-Celton, F-912130 Issy-les-Moulineaux, France; 9 University of Angers, Centre Hospitalier Universitaire d’Angers, Université de Rennes, Centre de consultations de pathologie professionnelle et santé au travail, F-49000 Angers, France; 10 AP-HP.Centre-Université de Paris, DMU Psychiatrie et Addictologie, Service de Psychiatrie de l’adulte, Hôpital Hôtel-Dieu, F-75004 Paris, France

**Keywords:** Addictology, Long working hours, Night shifts, Non-fixed working hours, Occupational health, Public health, Substance use, Workplace

## Abstract

**Background:**

This study examined prospective associations between atypical working hours with subsequent tobacco, cannabis and alcohol use as well as sugar and fat consumption.

**Methods:**

In the French population-based CONSTANCES cohort, 47,288 men and 53,324 women currently employed included between 2012 and 2017 were annually followed for tobacco and cannabis use. Among them, 35,647 men and 39,767 women included between 2012 and 2016 were also followed for alcohol and sugar and fat consumption. Three indicators of atypical working hours were self-reported at baseline: working at night, weekend work and non-fixed working hours. Generalized linear models computed odds of substance use and sugar and fat consumption at follow-up according to atypical working hours at baseline while adjusting for sociodemographic factors, depression and baseline substance use when appropriate.

**Results:**

Working at night was associated with decreased smoking cessation and increased relapse in women [odds ratios (ORs) of 0.81 and 1.25], increased cannabis use in men [ORs from 1.46 to 1.54] and increased alcohol use [ORs from 1.12 to 1.14] in both men and women. Weekend work was associated with decreased smoking cessation in women [ORs from 0.89 to 0.90] and increased alcohol use in both men and women [ORs from 1.09 to 1.14]. Non-fixed hours were associated with decreased smoking cessation in women and increased relapse in men [ORs of 0.89 and 1.13] and increased alcohol use in both men and women [ORs from 1.12 to 1.19]. Overall, atypical working hours were associated with decreased sugar and fat consumption.

**Conclusions:**

The potential role of atypical working hours on substance use should be considered by public health policy makers and clinicians in information and prevention strategies.

**Supplementary Information:**

The online version contains supplementary material available at 10.1186/s12889-022-14246-x.

## Introduction

Substance use are the first preventable cause of premature death worldwide [1]. If left untreated, they could lead to somatic disorders (e.g., cancers and cardiovascular disorders) [2, 3], psychiatric disorders (e.g., mood disorders and suicide) [4–7] and social deprivation including occupational issues (e.g., absenteeism, work accident and job loss) [8, 9]. Sugar and fat overconsumption are also highly prevalent in western countries, and they share common vulnerability factors with substance use [10].

Substance use and sugar and fat consumption could be driven by occupational factors [11]. For instance, work stress and high job demand may increase the likelihood of substance use and relapse in former users [12, 13]. The number of workers having atypical hours is increasing [14, 15]. Among the different types of atypical working hours, the following ones may be particularly frequent: working at night, working on weekend (i.e., Saturdays and/or Sundays) and having non-fixed schedules [16]. In this study, we focused on atypical working hours and their associations with tobacco, cannabis and alcohol use and high sugar and fat consumption. Such working conditions have already been associated with a broad range of somatic, psychiatric and sleep disorders, as well as increased risk of work accidents [17–29].

However, their potential consequences on substance use and sugar and fat consumption have not been examined yet, to the best of our knowledge. Since occupational health strategies exist to deal with atypical working hours at work, their benefits could be extended to decreasing the burden of such detrimental behaviors.

In a cross-sectional Spanish study on 3950 men and 3153 women aged 16–64 years, long working hours (i.e., 51–60 hours per week) were associated with higher odds of tobacco use in men and women compared to regular working hours [17]. In a meta-analysis conducted on alcohol use and long working hour, long working hours (i.e., more than 55 hours per week) were associated with alcohol use and new onset risky alcohol use in cross sectional studies [30]. Moreover, long working hours have been shown to be associated with time-related barriers to healthy eating, which in turn may be associated with unhealthy snacking and a higher sugar and fat consumption. For instance, in a cross-sectional study conducted on 2287 participants, working > 40 hours per week was associated with time-related barriers to healthful eating most among young adult men and among females working both part-time and > 40 hours per week [31]. In a longitudinal study, working at night was associated with higher odds of smoking among 488 male workers [32]. Regarding night work and nutrition patterns, some studies have reported frequent snack consumption and poorer diet quality [33, 34]. A study conducted among female nurses has shown that nurses with non-day shifts were more likely to have non-optimal eating behaviors which may contribute to an increased intake of saturated fat [35]. In addition, in a prospective study among airline workers, night shift was associated with higher percentage from total fat and saturated fats [36]. In a cross-sectional study on 3871 workers, those with permanent night work showed the highest odds of being overweight and having increased abdominal obesity [37, 38].

To our knowledge, no longitudinal study examined the association between atypical working hours and tobacco, cannabis, alcohol use as well as sugar and fat consumption in a large population-based sample of men and women, including a broad range of different atypical working hours (i.e., long working hours, working at night, non-fixed working hours) and while considering potential sociodemographic and clinical confounders. Hence, we took advantage of the French national population-based CONSTANCES cohort to examine prospectively the associations between atypical working hours and tobacco, cannabis, alcohol use and sugar and fat consumption in a large sample of workers from various social and occupational backgrounds [39]. Since patterns of substance use and occupational conditions usually differ according to sex, all these associations were examined in men and women separately [40, 41]. We hypothesized that atypical working hours would be associated with higher substance use and sugar and fat consumption.

## Methods

### Participants

The French population-based CONSTANCES cohort enrolled volunteers from 2012 to 2019, aged 18–69 years at baseline, according to a random sampling scheme stratified on age, gender, socioeconomic status, and region of France [39]. Among the different procedures conducted with participants, they completed annual self-administered questionnaires on their lifestyle, health, social, and personal characteristics. Additionally, they underwent physical examination in health-screening centers. The response rate at enrollment in the CONSTANCES cohort was of 7.3% [42], thus, in line with other international cohorts (e.g., 5.5% for the UK Biobank) [43]. All the procedures are detailed at www.constances.fr.

**Fig. 1:**
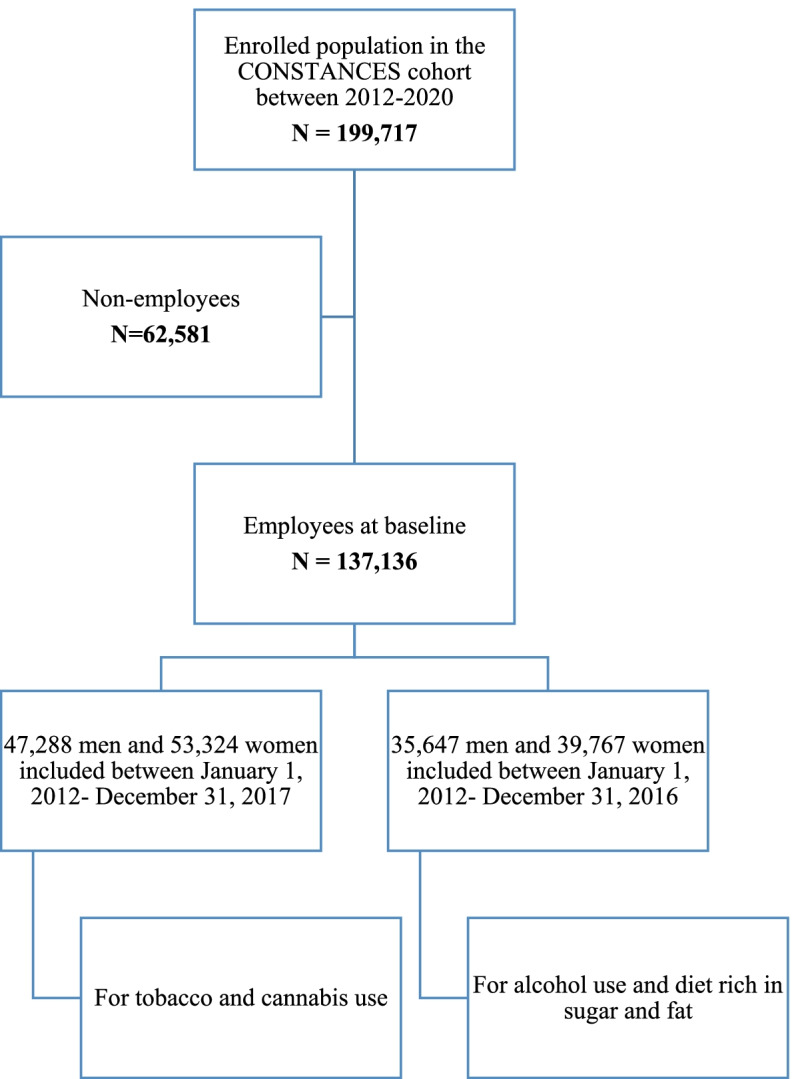
Cohort flow chart in the CONSTANCES cohort

The main CONSTANCES cohort consists of a total of 199,717 volunteers enrolled between January 6, 2012, and January 8, 2020. However, according to the present study’s aims, those who were not employed at baseline (*n* = 62, 581) were not included. In addition, since outcomes were available at different periods of follow-up, individuals included after January 1, 2018 (*n* = 36,524) were excluded when studying the tobacco and cannabis outcomes, to allow for one-year of follow-up duration (since the last follow-up date of these outcomes was in 2018 at the time the present study was conducted). Regarding alcohol and sugar and fat outcomes, volunteers included after January 2017 (*n* = 61,722) were excluded since the last available follow-up endpoint was in 2017 for these outcomes. Data on sugar and fat was available only at baseline and at follow-up in 2017. Hence, a total of 47,288 men and 53,324 women participants were included for studying tobacco and cannabis use. Among them, a total of 35,647 men and 39,767 women were included for studying alcohol use and sugar and fat consumption (Fig. 1 and Supplementary Table S1).

### Atypical working hours (exposures assessed at baseline)

Based on seven ‘Yes/No’ questions on atypical working hours that were analyzed separately, three different types of indicators were built.

First, night shifts were assessed based on the following questions: ‘Do you have (or have you had) work and travel times requiring you not to sleep at night for at least 50 days per year?’ and ‘Do you have (or have you had) work and travel times requiring you to go to bed after midnight for at least 50 days per year?’

Second, weekend work was assessed based on the following questions: ‘Do you work (or have you worked) more than one in two Sundays during the year?’ and ‘Do you work (or have you worked) more than one in two Saturdays during the year?’

Since the above questions were lifetime exposures to night shifts and week work and our main objective was to study the baseline atypical working hours, we selected only individuals who were currently exposed at baseline based on their date of exposure.

Third, non-fixed working hours were assessed based on the following questions that were only addressed to individuals who had a current job at baseline: ‘Do you work the same number of hours each day?’; ‘Do you work the same number of days each week?’ and ‘Do you work fixed hours?’

Answering “Yes” to any of the first four questions and “No” to any of the last three questions was considered as having a job with atypical working hours.

Even if we chose to give arbitrarily a label to identify three patterns of exposures to simplify the reading (i.e., “night shifts”, “weekend work”, “non-fixed working hours”), each exposure had to be studied separately since these exposures have been associated with different socio-occupational conditions and different health consequences, even within the same pattern [44–46].

### Substance use and diet rich in sugar and fat (outcomes assessed at follow-up)

#### Tobacco use

Since the initiation of tobacco use almost always preexist to adulthood [47, 48], we focused on changes in tobacco use at follow-up among ever users (i.e., being a former or a current user at baseline). Precisely, the following indicators were computed:

- Relapse of tobacco use among former smokers at baseline, i.e., reporting being a current smoker at follow-up while reporting being a former smoker at baseline.
- Changing smoking status at follow-up among ever smokers at baseline, defined as participants who were ex-smokers or current smokers at baseline, irrespective of their current or past level of consumption. Thus, this outcome had four categories as follows: current smokers at baseline and remained current smokers at follow-up (reference category), current smokers at baseline and stopped smoking at follow-up, ex-smokers at baseline and remained ex-smokers at follow-up and ex-smokers at baseline and relapsed at follow-up.

#### Cannabis use

Since the initiation of cannabis use almost always preexist to adulthood [48], we focused on ever-users (i.e., participants who reported having ever used cannabis during their lifetime at baseline). Due to restricted sample size compared to tobacco use, we computed only the following indicator in three categories reflecting cannabis use at follow-up: former user, cannabis user of less than once a month, cannabis user at least once per month.”

#### Alcohol use

Since becoming alcohol abstainers is a rare phenomenon at a population level at least in France [49], we focused only on alcohol consumption categories at follow-up based on the World Health Organization (WHO) risk level classification (World Health Organization, 2000) as follows: low risk (1–27 drinks/week in men and 1–13 in women), no use, and at risk (≥28 drinks/week in men and ≥ 14 in women).

### Diet rich in sugar and fat

Diet rich in sugar and fat was assessed using the 32-item qualitative food frequency questionnaire. This questionnaire was designed to reflect the intake in the French population and data regarding nutritional intake in the CONSTANCES cohort has already been published [50, 51]. The selected food items are compliant with the nutritional guidelines from the French National Nutrition and Health Program (PNNS) [52]. These items represented the weekly frequency of the consumed food (i.e., sugar, meat, cheese, yogurt, and others) on a scale from 0 to 4 with 0 being ‘never or nearly never’ and 4 ‘4 to 6 times per week’. Because food frequency was not normally distributed, each item’s square root was calculated and entered into a principal component analysis in order to identify and compute factors underlying the dietary patterns of the population [51]. Three factors were generated: diet rich in sugar and fat which was our variable of interest, traditional diet and diet rich in low fat protein (Supplementary Table S2). Diet rich in sugar and fat was assessed as quartiles variables: the first quartile which was the reference group corresponded to the lowest sugar and fat consumption and the fourth quartile to the highest consumption.

### Covariates at baseline

Sociodemographic factors included age, occupational grade (low: manual and clerical; medium: technical; high: managerial positions), educational level and household income. Educational level and household income were assessed using self-reported questions on the highest obtained diploma based on the International Standard Classification of Education 2011 [53], and on total household net monthly income, respectively. Since these two variables were ordinal representation of underlying sets of continuous units, they were used as continuous variables.

Depression was assessed using the presence of a treated depression as reported by the physician during the medical exam at inclusion and treated as a binary variable (‘Yes’ versus ‘No’).

### Statistical analysis

Generalized linear regressions were computed to study the associations between the indicators of atypical working hours (exposures) and tobacco, cannabis, alcohol use and diet rich in sugar and fat (outcomes). In other terms, binary logistic regressions were computed to study the associations between these indicators and relapse of tobacco use. Multinominal logistic regressions were computed to study the association between the exposures and sugar and fat intake as well as changing statuses in tobacco use, cannabis relapse. The associations between these indicators, tobacco and cannabis were studied until 2018 which was the last follow-up endpoint. Whereas the associations between these indicators, alcohol and diet rich in sugar and fat were studied until the last available follow-up endpoint which was in 2017. All the analyses were stratified by sex.

After computing univariable analyses, fully-adjusted models were performed including all the covariables mentioned above in addition to the baseline level of consumption for the substance chosen as the outcome. Regarding the baseline level of substance consumption, we adjusted for it for alcohol use and diet rich in sugar and fat. However, we did not adjust for it for tobacco and cannabis use since this variable was already included in the outcomes.

Sensitivity analyses were performed as supplementary analyses:

- First, since job type could be associated with the aforementioned outcomes, we tested for statistical interactions between occupational grade and indicators of atypical working hours. We further examined the association between these indicators and outcomes in stratified analyses according to occupations. Occupations were categorized in four groups: ‘Farmers, blue-collar workers and craftsmen’; ‘Clerks’; ‘Intermediate workers’ and ‘Executives’.
- Second, since the associations between each atypical working hours indicator and fat and sugar dietary patterns may be more pronounced among individuals with a lifestyle involving specific eating behaviors (e.g., sedentary lifestyle, currently on a diet, high physical activity), interactions between atypical working hours and BMI (< 25; ≥25 and < 30; ≥30), physical activity (score from 1 to 6, 0: not active and 6: very active) and being currently on diet (‘Yes’; ‘No’) were tested.
- Third, since duration of exposure could play a role in the associations between atypical working hours and substance, theses associations were stratified by duration of exposure when information was available (i.e., the exposures related to night shifts (‘do you have (or have you had) work and travel times requiring you not to sleep at night for at least 50 days per year?’; ‘do you have (or have you had) work and travel times requiring you to go to bed after midnight for at least 50 days per year?’) and weekend work (‘do you work (or have you worked) more than one in two Saturdays during the year?’; ‘do you work (or have you worked) more than one in two Sundays during the year?’)). To measure the duration of exposure, we used the difference between the first date of exposure and the last date of exposure.

Missing data were handled by multiple imputations [54].

All *p*-values were two-sided with an α = 0.05. All statistical analyses were undertaken using the SAS system software (version 9.4, SAS Institute, Cary, NC).

## Results

The baseline characteristics of the 47,288 men and 53,324 women included in 2012–2017, and of the 35,647 men and 39,767 women included in 2012–2016 are summarized in Table 1.

**Table 1:** Baseline characteristics of men and women included between 2012 and 2017 and 2012–2016, respectively in the CONSTANCES cohort

	Between 2012 and 2017		Between 2012 and 2016	
	Men	Women	Men	Women
	*N* = 47,288	*N* = 53,324	*N* = 35,647	*N* = 39,767
Sociodemographic and clinical factors				
Mean (SD) age, years	43.8 (10.9)	43.3 (10.9)	44.2 (10.9)	43.6 (10.9)
Occupational grade, %				
Low	34.8	41.0	34.0	40.6
Medium	25.2	32.5	25.5	32.7
High	40.0	26.5	40.5	26.7
Educational level using the 2011 ISCED, %				
Levels 0 to 1	3.0	2.1	3.0	2.1
Level 2	3.6	3.7	3.7	4.0
Levels 3 to 4	32.4	27.4	32.7	27.9
Levels 5 to 6	31.3	41.9	31.2	41.7
Levels 7 to 8	29.7	24.9	29.4	24.3
Household income in euros per month, %				
< 2100	17.9	22.2	17.6	22.4
2100–2800	14.6	16.1	14.8	16.2
2800–4200	33.1	32.8	32.5	32.5
> 4200	34.4	28.9	35.0	28.9
Depression^a^, %	9.6	17.8	9.9	18.1
Indicators of atypical working hours				
Do you have (or have you had) work and travel times requiring you not to sleep at night at least 50 days/year? -Yes, %	9.7	6.2	9.7	5.4
Do you have (or have you had) work and travel times requiring you to go to bed after midnight at least 50 days/year? -Yes, %	15.1	8.3	15.2	8.3
Do you work (or have you worked) more than one in two Sundays during the year? -Yes, %	13.2	14.5	13.2	14.4
Do you work (or have you worked) more than one in two Saturdays during the year? -Yes, %	25.5	28.4	25.5	28.3
Do you work the same number of hours each day? -No, %	47.5	49.7	46.9	49.2
Do you work the same number of days each week? -No, %	23.2	24.4	23.2	24.0
Do you work fixed hours? -No, %	42.5	36.0	42.0	35.8

^a^Depression was assessed at baseline using the presence of a treated depression

*ISCED* International Standard Classification of Education

Compared to workers that were not exposed to atypical working hours, both men and women with atypical working hours were older, had a higher prevalence of low occupational grade and had a lower prevalence of high education or income. Depression was associated with several indicators of atypical working hours with more frequent associations in men than in women (Supplementary Tables S3, S4, S5 and S6).

### Association between working at night, substance use and diet rich in sugar and fat (Table 2)

#### Tobacco use

Working after midnight was associated with increased odds of relapse in women that were former smokers (adjusted odds ratio (aOR): 1.25, 95%CI: 1.09–1.43). In women that were current smokers at baseline, both working all night and after midnight were associated with decreased odds of quitting (aOR: 0.86, 95% CI: 0.77–0.96 and aOR: 0.78, 95% CI: 0.72–0.84, respectively).

**Table 2:** Associations between two exposures regarding night work, substance use and diet

	Men			Women		
		Unadjusted model	Fully-adjusted model^a^		Unadjusted model	Fully-adjusted model^a^
	N (%)	OR (95% CI)	OR (95% CI)	N (%)	OR (95% CI)	OR (95% CI)
**Do you have (or have you had) work and travel times requiring you not to sleep at night at least 50 days/year?**						
**Tobacco use**						
Relapse of tobacco use among ex-smokers at baseline	15,452			15,464		
No	12,483 (80.8)	1.00	1.00	12,735 (82.4)	1.00	1.00
Yes	2969 (19.2)	**1.20 (1.06–1.36)**	1.02 (0.90–1.15)	2729 (17.6)	1.17 (0.98–1.39)	1.21 (0.99–1.42)
Changing status among ever-smokers at baseline	25,402			25,592		
Smokers at baseline and remained current smokers at follow-up	7145 (28.2)	1.00	1.00	7146 (27.9)	1.00	1.00
Smokers at baseline and stopped at follow-up	2805 (11.0)	**0.71 (0.61–0.82)**	0.93 (0.81–1.07)	2982 (11.6)	**0.80 (0.66–0.96)**	0.85 (0.72–1.01)
Ex-smokers at baseline and remained ex-smokers at follow-up	12,483 (49.1)	**0.85 (0.78–0.93)**	1.00 (0.91–1.09)	12,735 (49.8)	**0.80 (0.71–0.90)**	**0.86 (0.77–0.96)**
Ex-smokers at baseline and relapsed at follow-up	2969 (11.7)	1.02 (0.90–1.17)	1.03 (0.91–1.16)	2729 (10.7)	0.93 (0.78–1.12)	1.03 (0.88–1.21)
**Cannabis use**						
Cannabis use among ever-users at baseline	17,924			16,304		
No use in the past 12 months	16,817 (93.8)	1.00	1.00	15,514 (95.1)	1.00	1.00
Use in the past 12 months, < 1/month	930 (5.2)	0.82 (0.64–1.04)	0.91 (0.73–1.14)	628 (3.9)	1.20 (0.87–1.64)	1.12 (0.83–1.50)
Use in the past 12 months, ≥1/month	177 (1.0)	**1.54 (1.02–2.34)**	**1.54 (1.07–2.23)**	162 (1.0)	0.59 (0.26–1.34)	0.55 (0.26–1.19)
**Alcohol use (WHO risk levels)**	35,647			39,767		
Low risk	27,554 (77.3)	1.00	1.00	22,246 (55.9)	1.00	1.00
No use	4977 (14.0)	**1.28 (1.16–1.41)**	**1.13 (1.03–1.24)**	10,785 (27.1)	**1.17 (1.06–1.29)**	1.04 (0.95–1.14)
At risk	3116 (8.7)	1.09 (0.96–1.23)	0.99 (0.87–1.12)	6736 (17.0)	0.93 (0.82–1.06)	0.94 (0.84–1.06)
**Diet rich in sugar and fat**	35,647			39,767		
First quartile	8842 (24.8)	1.00	1.00	9861 (24.8)	1.00	1.00
Second quartile	8981 (25.2)	**0.98 (0.89–1.08)**	0.95 (0.87–1.04)	10,022 (25.2)	0.95 (0.84–1.08)	0.96 (0.86–1.08)
Third quartile	8912 (25.0)	0.91 (0.83–1.01)	0.92 (0.84–1.01)	9942 (25.0)	0.99 (0.88–1.12)	1.07 (0.95–1.19)
Fourth quartile	8912 (25.0)	**0.79 (0.72–0.88)**	**0.86 (0.78–0.95)**	9942 (25.0)	0.99 (0.88–1.12)	1.02 (0.91–1.14)
**Do you have (or have you had) work and travel times requiring you to go to bed after midnight at least 50 days/year?**						
**Tobacco use**						
Relapse of tobacco use among ex-smokers at baseline	15,452			15,464		
No	12,483 (80.8)	1.00	1.00	12,735 (82.4)	1.00	1.00
Yes	2969 (19.2)	**1.33 (1.20–1.48)**	1.08 (0.96–1.21)	2729 (17.6)	**1.39 (1.21–1.60)**	**1.25 (1.09–1.43)**
Changing status among ever-smokers at baseline	25,402			25,592		
Smokers at baseline and remained current smokers at follow-up	7145 (28.2)	1.00	1.00	7146 (27.9)	1.00	1.00
Smokers at baseline and stopped at follow-up	2805 (11.0)	**0.83 (0.74–0.94)**	0.92 (0.83–1.02)	2982 (11.6)	**0.79 (0.69–0.91)**	**0.81 (0.72–0.91)**
Ex-smokers at baseline and remained ex-smokers at follow-up	12,483 (49.1)	**0.77 (0.71–0.83)**	**0.90 (0.84–0.97)**	12,735 (49.8)	**0.64 (0.58–0.71)**	**0.78 (0.72–0.84)**
Ex-smokers at baseline and relapsed at follow-up	2969 (11.7)	1.02 (0.91–1.14)	1.01 (0.92–1.11)	2729 (10.7)	0.89 (0.77–1.03)	0.98 (0.87–1.10)
**Cannabis use**						
Cannabis use among ever-users at baseline	17,924			16,304		
No use in the past 12 months	16,817 (93.8)	1.00	1.00	15,514 (95.1)	1.00	1.00
Use in the past 12 months, < 1/month	930 (5.2)	0.87 (0.72–1.05)	1.06 (0.90–1.25)	628 (3.9)	**1.67 (1.34–2.10)**	**1.32 (1.08–1.61)**
Use in the past 12 months, ≥1/month	177 (1.0)	**1.46 (1.03–2.08)**	**1.40 (1.02–1.91)**	162 (1.0)	1.29 (0.81–2.07)	0.88 (0.58–1.34)
**Alcohol use (WHO risk levels)**	35,647			39,767		
Low risk	27,554 (77.3)	1.00	1.00	22,246 (55.9)	1.00	1.00
No use	4977 (14.0)	**1.11 (1.02–1.20)**	1.04 (0.96–1.12)	10,785 (27.1)	1.06 (0.97–1.15)	0.97 (0.90–1.04)
At risk	3116 (8.7)	**1.22 (1.11–1.35)**	**1.12 (1.02–1.24)**	6736 (17.0)	**1.25 (1.13–1.37)**	**1.14 (1.05–1.24)**
**Diet rich in sugar and fat**	35,647			39,767		
First quartile	8842 (24.8)	1.00	1.00	9861 (24.8)	1.00	1.00
Second quartile	8981 (25.2)	0.95 (0.88–1.03)	0.94 (0.87–1.01)	10,022 (25.2)	0.94 (0.85–1.04)	0.95 (0.88–1.03)
Third quartile	8912 (25.0)	0.98 (0.90–1.06)	0.96 (0.89–1.03)	9942 (25.0)	0.94 (0.85–1.04)	0.94 (0.87–1.02)
Fourth quartile	8912 (25.0)	**0.88 (0.81–0.96)**	**0.91 (0.95–0.98)**	9942 (25.0)	0.99 (0.90–1.09)	**0.91 (0.83–0.99)**

^a^Adjusted for age (years, continuous), occupational grade (low; medium; high), educational level (levels of based on the 2011 International Standard Classification of Education, continuous), household income (€/month, continuous) and baseline depression (yes; no). Adjustments for level of consumption at baseline were performed for alcohol use and diet rich in sugar and fat

Relapse was defined as: no (remained non-smokers at follow-up) and yes (became current smokers at follow-up)

World Health Organization (WHO) risk level classification were used as follows: low risk (1–27 drinks/week in men and 1–13 in women), no use, and at risk (≥28 drinks/week in men and ≥ 14 in women)

#### Cannabis use

In men who were not cannabis users for the last 12 months, working all night and after midnight were associated with increased odds of using cannabis at least once per month at follow-up (aOR: 1.54, 95% CI: 1.07–2.23 and aOR:1.40, 95%CI: 1.02–1.91, respectively).

#### Alcohol use

Working after midnight was associated with increased odds of alcohol use in both women and men (aOR: 1.14, 95%CI: 1.05–1.24 and aOR:1.12, 95%CI: 1.02–1.91, respectively).

### Diet rich in sugar and fat

Working all night was associated with a decreased odd of consuming a diet rich in sugar and fat in men (aOR:0.86, 95%CI: 0.78–0.95 for the fourth quartile compared to the first). Similar results were found for working after midnight in both men and women (aOR: 0.91, 95%CI: 0.95–0.98 and aOR: 0.91, 95%CI: 0.83–0.99, respectively).

Association between weekend work, substance use and diet rich in sugar and fat (Table 3).

**Table 3:** Associations between two exposures regarding weekend work, substance use and diet

	Men			Women		
		Unadjusted model	Fully-adjusted model^a^		Unadjusted model	Fully-adjusted model^a^
	N (%)	OR (95% CI)	OR (95% CI)	N (%)	OR (95% CI)	OR (95% CI)
**Do you work (or have you worked) more than one in two Sundays during the year?**						
**Tobacco use**						
Relapse of tobacco use among ex-smokers at baseline	15,452			15,464		
No	12,483 (80.8)	1.00	1.00	12,735 (82.4)	1.00	1.00
Yes	2969 (19.2)	**1.32 (1.18–1.48)**	1.07 (0.96–1.19)	2729 (17.6)	**1.16 (1.04–1.30)**	1.00 (0.90–1.11)
Changing status among ever-smokers at baseline	25,402			25,592		
Smokers at baseline and remained current smokers at follow-up	7145 (28.2)	1.00	1.00	7146 (27.9)	1.00	1.00
Smokers at baseline and stopped at follow-up	2805 (11.0)	**0.73 (0.64–0.83)**	0.96 (0.86–1.06)	2982 (11.6)	**0.66 (0.59–0.74)**	**0.89 (0.80–0.99)**
Ex-smokers at baseline and remained ex-smokers at follow-up	12,483 (49.1)	**0.75 (0.69–0.81)**	0.95 (0.88–1.02)	12,735 (49.8)	**0.68 (0.63–0.74)**	**0.92 (0.85–0.99)**
Ex-smokers at baseline and relapsed at follow-up	2969 (11.7)	0.99 (0.88–1.11)	1.00 (0.91–1.11)	2729 (10.7)	**0.79 (0.71–0.89)**	0.91 (0.82–1.02)
**Cannabis use**						
Cannabis use among ever-users at baseline	17,924			16,304		
No use in the past 12 months	16,817 (93.8)	1.00	1.00	15,514 (95.1)	1.00	1.00
Use in the past 12 months, < 1/month	930 (5.2)	0.88 (0.72–1.08)	0.95 (0.80–1.12)	628 (3.9)	1.15 (0.93–1.43)	1.11 (0.91–1.35)
Use in the past 12 months, ≥1/month	177 (1.0)	1.18 (0.78–1.76)	1.16 (0.84–1.61)	162 (1.0)	**1.67 (1.16–2.42)**	1.00 (0.67–1.44)
**Alcohol use (WHO risk levels)**	35,647			39,767		
Low risk	27,554 (77.3)	1.00	1.00	22,246 (55.9)	1.00	1.00
No use	4977 (14.0)	**1.31 (1.20–1.42)**	**1.09 (1.01–1.18)**	10,785 (27.1)	**1.20 (1.12–1.28)**	1.02 (0.96–1.08)
At risk	3116 (8.7)	**1.22 (1.10–1.35)**	1.06 (0.96–1.17)	6736 (17.0)	**1.19 (1.10–1.28)**	**1.09 (1.02–1.18)**
**Diet rich in sugar and fat**	35,647			39,767		
First quartile	8842 (24.8)	1.00	1.00	9861 (24.8)	1.00	1.00
Second quartile	8981 (25.2)	**0.81 (0.75–0.89)**	**0.89 (0.83–0.96)**	10,022 (25.2)	0.93 (0.86–1.01)	0.98 (0.91–1.05)
Third quartile	8912 (25.0)	**0.84 (0.77–0.92)**	**0.88 (0.82–0.95)**	9942 (25.0)	**0.89 (0.83–0.97)**	0.93 (0.85–1.01)
Fourth quartile	8912 (25.0)	**0.77 (0.70–0.84)**	**0.80 (0.75–0.87)**	9942 (25.0)	0.93 (0.86–1.01)	1.00 (0.93–1.08)
**Do you work (or have you worked) more than one in two Saturdays during the year?**						
**Tobacco use**						
Relapse of tobacco use among ex-smokers at baseline	15,452			15,464		
No	12,483 (80.8)	1.00	1.00	12,735 (82.4)	1.00	1.00
Yes	2969 (19.2)	**1.26 (1.15–1.37)**	1.08 (0.97–1.20)	2729 (17.6)	**1.14 (1.04–1.24)**	1.04 (0.94–1.15)
Changing status among ever-smokers at baseline	25,402			25,592		
Smokers at baseline and remained current smokers at follow-up	7145 (28.2)	1.00	1.00	7146 (27.9)	1.00	1.00
Smokers at baseline and stopped at follow-up	2805 (11.0)	**0.71 (0.64–0.78)**	0.91 (0.83–1.01)	2982 (11.6)	**0.75 (0.69–0.83)**	**0.90 (0.82–0.97)**
Ex-smokers at baseline and remained ex-smokers at follow-up	12,483 (49.1)	**0.74 (0.69–0.79)**	**0.92 (0.86–0.99)**	12,735 (49.8)	**0.72 (0.67–0.76)**	**0.93 (0.87–0.98)**
Ex-smokers at baseline relapsed at follow-up	2969 (11.7)	0.93 (0.85–1.02)	1.00 (0.91–1.10)	2729 (10.7)	**0.82 (0.74–0.90)**	0.96 (0.87–1.06)
**Cannabis use**						
Cannabis use among ever-users at baseline	17,924			16,304		
No use in the past 12 months	16,817 (93.8)	1.00	1.00	15,514 (95.1)	1.00	1.00
Use in the past 12 months, < 1/month	930 (5.2)	**0.81 (0.69–0.95)**	0.93 (0.80–1.09)	628 (3.9)	1.19 (0.90–1.34)	1.10 (0.92–1.32)
Use in the past 12 months, ≥1/month	177 (1.0)	1.35 (0.99–1.88)	1.34 (0.98–1.83)	162 (1.0)	1.33 (0.95–1.86)	1.00 (0.71–1.41)
**Alcohol use (WHO risk levels)**	35,647			39,767		
Low risk	27,554 (77.3)	1.00	1.00	22,246 (55.9)	**1.00**	1.00
No use	4977 (14.0)	**1.28 (1.20–1.37)**	**1.11 (1.03–1.19)**	10,785 (27.1)	**1.14 (1.08–1.19)**	1.03 (0.97–1.09)
At risk	3116 (8.7)	**1.27 (1.17–1.37)**	**1.13 (1.03–1.24)**	6736 (17.0)	**1.19 (1.12–1.26)**	**1.14 (1.07–1.22)**
**Diet rich in sugar and fat**	35,647			39,767		
First quartile	8842 (24.8)	1.00	1.00	9861 (24.8)	1.00	1.00
Second quartile	8981 (25.2)	**0.89 (0.84–0.95)**	**0.89 (0.83–0.95)**	10,022 (25.2)	0.94 (0.89–1.01)	**0.92 (0.87–0.99)**
Third quartile	8912 (25.0)	**0.89 (0.83–0.95)**	0.91 (0.85–0.98)	9942 (25.0)	0.94 (0.88–1.01)	0.93 (0.88–1.00)
Fourth quartile	8912 (25.0)	**0.79 (0.74–0.85)**	**0.85 (0.80–0.92)**	9942 (25.0)	0.95 (0.90–1.01)	**0.94 (0.88–0.99)**

^a^Adjusted for age (years, continuous), occupational grade (low; medium; high), educational level (levels, continuous), household income (€/month, continuous) and baseline depression (yes; no). Adjustments for level of consumption at baseline were performed for alcohol use and diet rich in sugar and fat

Relapse was defined as: no (remained non-smokers at follow-up) and yes (became current smokers at follow-up)

World Health Organization (WHO) risk level classification were used as follows: low risk (1–27 drinks/week in men and 1–13 in women), no use, and at risk (≥28 drinks/week in men and ≥ 14 in women)

#### Tobacco use

In women that are current smokers at baseline, Sunday work was associated with decreased odds of quitting (aOR: 0.89, 95% CI: 0.80–0.99). In both women and women, Saturday work was associated with decreased odds of quitting (aOR: 0.93, 95%CI: 0.87–0.98 and aOR: 0.92, 95%CI: 0.86–0.99, respectively).

#### Cannabis use

No significant association was found between weekend work and cannabis use.

#### Alcohol use

In women, Sunday work was associated with increased odds of alcohol use (aOR: 1.09, 95%CI: 1.02–1.18).

Saturday work was associated with increased odds of alcohol use in both women and men (aOR: 1.14, 95%CI: 1.07–1.22 and aOR: 1.13, 95%CI: 1.03–1.24, respectively).

### Diet rich in sugar and fat

Sunday work was associated with a decreased odd of consuming a diet rich in sugar and fat in men (aOR:0.80, 95%CI: 0.75–0.87 for the fourth quartile compared to the first). Similar results were found for Saturday work in both men and women (aOR: 0.85, 95%CI: 0.80–0.92 and aOR: 0.94, 95%CI: 0.80–0.99, respectively).

Association between non-fixed working hours, substance use and diet rich in sugar and fat (Table 4).

**Table 4:** Associations between three exposures regarding non-fixed working hours, substance use and diet

	Men			Women		
		Unadjusted model	Fully-adjusted model^a^		Unadjusted model	Fully-adjusted model^a^
	N (%)	OR (95% CI)	OR (95% CI)	N (%)	OR (95% CI)	OR (95% CI)
**Do you work the same number of hours each day?**						
**Tobacco use**						
Relapse of tobacco use among ex-smokers at baseline	15,452			15,464		
No	12,483 (80.8)	1.00	1.00	12,735 (82.4)	1.00	1.00
Yes	2969 (19.2)	1.00 (0.92–1.08)	1.08 (0.99–1.19)	2729 (17.6)	1.05 (0.96–1.14)	1.07 (0.98–1.17)
Changing status among ever-smokers at baseline	25,402			25,592		
Smokers at baseline and remained current smokers at follow-up	7145 (28.2)	1.00	1.00	7146 (27.9)	1.00	1.00
Smokers at baseline and stopped at follow-up	2805 (11.0)	**1.12 (1.02–1.22)**	0.97 (0.89–1.07)	2982 (11.6)	0.97 (0.89–1.06)	**0.89 (0.81–0.97)**
Ex-smokers at baseline and remained ex-smokers at follow-up	12,483 (49.1)	**0.90 (0.85–0.95)**	**0.83 (0.78–0.89)**	12,735 (49.8)	**0.94 (0.89–0.99)**	**0.92 (0.86–0.98)**
Ex-smokers at baseline and relapsed at follow-up	2969 (11.7)	**0.90 (0.83–0.98)**	**0.90 (0.82–0.98)**	2729 (10.7)	0.99 (0.90–1.08)	0.99 (0.91–1.09)
**Cannabis use**						
Cannabis use among ever-users at baseline	17,924			16,304		
No use in the past 12 months	16,817 (93.8)	1.00	1.00	15,514 (95.1)	1.00	1.00
Use in the past 12 months, < 1/month	930 (5.2)	**1.21 (1.01–1.38)**	1.16 (1.02–1.33)	628 (3.9)	1.18 (0.95–1.47)	1.17 (0.94–1.47)
Use in the past 12 months, ≥1/month	177 (1.0)	0.90 (0.67–1.23)	0.97 (0.72–1.29)	162 (1.0)	1.18 (0.74–1.87)	1.23 (0.76–1.97)
**Alcohol use (WHO risk levels)**	35,647			39,767		
Low risk	27,554 (77.3)	1.00	1.00	22,246 (55.9)	1.00	1.00
No use	4977 (14.0)	**0.92 (0.88–0.96)**	1.02 (0.97–1.07)	10,785 (27.1)	0.96 (0.92–1.00)	1.03 (0.98–1.08)
At risk	3116 (8.7)	**1.16 (1.06–1.26)**	**1.15 (1.05–1.26)**	6736 (17.0)	**1.21 (1.13–1.30)**	**1.14 (1.06–1.23)**
**Diet rich in sugar and fat**	35,647			39,767		
First quartile	8842 (24.8)	1.00	1.00	9861 (24.8)	1.00	1.00
Second quartile	8981 (25.2)	1.00 (0.92–1.09)	0.95 (0.83–1.10)	10,022 (25.2)	0.95 (0.87–1.03)	0.89 (0.78–1.03)
Third quartile	8912 (25.0)	0.96 (0.88–1.05)	0.87 (0.75–1.02)	9942 (25.0)	0.97 (0.89–1.05)	0.99 (0.85–1.15)
Fourth quartile	8912 (25.0)	1.02 (0.93–1.11)	0.88 (0.75–1.05)	9942 (25.0)	1.00 (0.92–1.09)	0.98 (0.83–1.16)
**Do you work the same number of days each week?**						
**Tobacco use**						
Relapse of tobacco use among ex-smokers at baseline	15,452			15,464		
No	12,483 (80.8)	1.00	1.00	12,735 (82.4)	1.00	1.00
Yes	2969 (19.2)	**1.18 (1.07–1.29)**	**1.13 (1.01–1.27)**	2729 (17.6)	**1.12 (1.01–1.23)**	1.05 (0.95–1.16)
Changing status among ever-smokers at baseline	25,402			25,592		
Smokers at baseline and remained current smokers at follow-up	7145 (28.2)	1.00	1.00	7146 (27.9)	1.00	1.00
Smokers at baseline and stopped at follow-up	2805 (11.0)	0.95 (0.86–1.05)	1.06 (0.95–1.18)	2982 (11.6)	**0.88 (0.80–0.97)**	0.91 (0.82–1.01)
Ex-smokers at baseline and remained ex-smokers at follow-up	12,483 (49.1)	**0.80 (0.75–0.86)**	**0.87 (0.81–0.94)**	12,735 (49.8)	**0.79 (0.74–0.84)**	**0.90 (0.84–0.97)**
Ex-smokers at baseline and relapsed at follow-up	2969 (11.7)	0.94 (0.86–1.04)	0.98 (0.88–1.08)	2729 (10.7)	**0.88 (0.80–0.97)**	0.96 (0.86–1.06)
**Cannabis use**						
Cannabis use among ever-users at baseline	17,924			16,304		
No use in the past 12 months	16,817 (93.8)	1.00	1.00	15,514 (95.1)	1.00	1.00
Use in the past 12 months, < 1/month	930 (5.2)	0.95 (0.81–1.11)	0.99 (0.84–1.16)	628 (3.9)	1.09 (0.86–1.37)	1.03 (0.80–1.32)
Use in the past 12 months, ≥1/month	177 (1.0)	1.16 (0.84–1.61)	1.05 (0.76–1.46)	162 (1.0)	1.49 (0.92–1.68)	1.38 (0.84–1.66)
**Alcohol use (WHO risk levels)**	35,647			39,767		
Low risk	27,554 (77.3)	1.00	1.00	22,246 (55.9)	1.00	1.00
No use	4977 (14.0)	**1.15 (1.09–1.21)**	1.02 (0.97–1.09)	10,785 (27.1)	**1.12 (1.07–1.18)**	**1.07 (1.01–1.13)**
At risk	3116 (8.7)	**1.32 (1.20–1.46)**	**1.19 (1.06–1.32)**	6736 (17.0)	**1.14 (1.06–1.24)**	**1.12 (1.02–1.22)**
**Diet rich in sugar and fat**	35,647			39,767		
First quartile	8842 (24.8)	1.00	1.00	9861 (24.8)	1.00	1.00
Second quartile	8981 (25.2)	0.91 (0.82–1.10)	0.92 (0.78–1.08)	10,022 (25.2)	0.91 (0.82–1.00)	**0.81 (0.67–0.96)**
Third quartile	8912 (25.0)	**0.87 (0.79–0.97)**	0.87 (0.73–1.04)	9942 (25.0)	**0.91 (0.82–0.99)**	0.90 (0.75–1.07)
Fourth quartile	8912 (25.0)	**0.89 (0.80–0.99)**	0.85 (0.69–1.03)	9942 (25.0)	1.01 (0.92–1.12)	1.00 (0.82–1.21)
**Do you work fixed hours?**						
**Tobacco use**						
Relapse of tobacco use among ex-smokers at baseline	15,452			15,464		
No	12,483 (80.8)	1.00	1.00	12,735 (82.4)	1.00	1.00
Yes	2969 (19.2)	1.05 (0.97–1.14)	1.11 (0.95–1.30)	2729 (17.6)	1.03 (0.95–1.13)	1.05 (0.95–1.17)
Changing status among ever-smokers at baseline	25,402			25,592		
Smokers at baseline and remained current smokers at follow-up	7145 (28.2)	1.00	1.00	7146 (27.9)	1.00	1.00
Smokers at baseline and stopped at follow-up	2805 (11.0)	**1.16 (1.06–1.26)**	0.97 (0.88–1.07)	2982 (11.6)	0.99 (0.91–1.09)	0.92 (0.84–1.01)
Ex-smokers at baseline and remained ex-smokers at follow-up	12,483 (49.1)	**0.94 (0.88–0.99)**	**0.82 (0.77–0.88)**	12,735 (49.8)	0.95 (0.90–1.01)	0.93 (0.87–1.00)
Ex-smokers at baseline and relapsed at follow-up	2969 (11.7)	0.98 (0.90–1.07)	0.96 (0.87–1.05)	2729 (10.7)	0.99 (0.90–1.08)	1.00 (0.91–1.10)
**Cannabis use**						
Cannabis use among ever-users at baseline	17,924			16,304		
No use in the past 12 months	16,817 (93.8)	1.00	1.00	15,514 (95.1)	1.00	1.00
Use in the past 12 months, < 1/month	930 (5.2)	1.05 (0.92–1.20)	0.98 (0.85–1.12)	628 (3.9)	1.08 (0.86–1.34)	1.04 (0.88–1.28)
Use in the past 12 months, ≥1/month	177 (1.0)	0.72 (0.53–0.97)	0.80 (0.59–1.08)	162 (1.0)	1.10 (0.80–1.49)	1.09 (0.79–1.49)
**Alcohol use (WHO risk levels)**	35,647			39,767		
Low risk	27,554 (77.3)	1.00	1.00	22,246 (55.9)	1.00	1.00
No use	4977 (14.0)	**0.89 (0.85–0.93)**	1.04 (0.99–1.09)	10,785 (27.1)	**0.93 (0.89–0.97)**	1.00 (0.95–1.05)
At risk	3116 (8.7)	1.09 (0.99–1.19)	**1.15 (1.04–1.26)**	6736 (17.0)	**1.14 (1.06–1.22)**	1.05 (0.97–1.14)
**Diet rich in sugar and fat**	35,647			39,767		
First quartile	8842 (24.8)	1.00	1.00	9861 (24.8)	1.00	1.00
Second quartile	8981 (25.2)	1.00 (0.92–1.09)	1.06 (0.91–1.23)	10,022 (25.2)	0.97 (0.89–1.06)	0.91 (0.79–1.05)
Third quartile	8912 (25.0)	0.94 (0.86–1.03)	1.04 (0.89–1.21)	9942 (25.0)	0.99 (0.91–1.07)	1.01 (0.86–1.19)
Fourth quartile	8912 (25.0)	1.01 (0.93–1.11)	1.16 (0.97–1.38)	9942 (25.0)	0.99 (0.91–1.07)	1.03 (0.86–1.22)

^a^Adjusted for age (years, continuous), occupational grade (low; medium; high), educational level (levels, continuous), household income (€/month, continuous) and baseline depression (yes; no). Adjustments for level of consumption at baseline were performed for alcohol use and diet rich in sugar and fat

Relapse was defined as: no (remained non-smokers at follow-up) and yes (became current smokers at follow-up)

World Health Organization (WHO) risk level classification were used as follows: low risk (1–27 drinks/week in men and 1–13 in women), no use, and at risk (≥28 drinks/week in men and ≥ 14 in women)

#### Tobacco use

In current smokers at baseline, fluctuating number of working hours and working days were associated with decreased odds of quitting in both men and women (aOR: 0.83, 95% CI: 0.78–0.89 and aOR: 0.92, 95% CI: 0.86–0.98; aOR: 0.87, 95% CI: 0.81–0.94 and aOR: 0.90, 95% CI: 0.84–0.97, respectively).

#### Cannabis use

No significant association was found between non-fixed working hours and cannabis use.

#### Alcohol use

Fluctuating number of working hours and working days were associated with increased odds of alcohol use in both men and women (aOR: 1.15, 95%CI: 1.05–1.26 and aOR: 1.14, 95%CI: 1.06–1.23; aOR: 1.19, 95%CI: 1.06–1.32 and aOR: 1.12, 95%CI: 1.02–1.22, respectively).

### Diet rich in sugar and fat

No significant associations between non-fixed working hours and diet rich in sugar and fat were found.

### Sensitivity analyses

The stratified analyses showed that the associations between atypical working hours and substance use were more pronounced in workers from low occupational grade compared to those from high occupational grade. In addition, most of these associations persisted in workers exposed since less than 1 year compared to individuals exposed since at least 1 year (data not shown).

No interactions were found between atypical working hours and BMI, physical activity or following a current diet when examining the associations between atypical working hours and diet rich in fat or sugar (data not shown**)**.

## Discussion

This study examined the prospective associations between atypical working hours at work and tobacco, cannabis, alcohol use, and a diet rich in sugar and fat among workers from a large population-based cohort while taking into account sociodemographic factors and depression. Overall, working at night was associated with decreased smoking cessation and increased relapse in women, increased cannabis use in men and increased alcohol use in both men and women. Weekend work was associated with decreased smoking cessation in women and increased alcohol use in both men and women. Non-fixed hours was associated with decreased smoking cessation in women and increased relapse in men and increased alcohol use in both men and women. Overall, atypical working hours were associated with decreased sugar and fat consumption.

This study has some strengths. First, the CONSTANCES cohort is a national population-based cohort from various sociodemographic and occupational conditions [39]. Second, we had the necessary data to adjust the analyses for potential confounders, and sufficient power to run stratified analyses by sex. Third, we had different questions to assess atypical working hours and the use of several substances and dietary intake. However, this study has some limitations. First, although the CONSTANCES cohort is a large population-based cohort with different work settings, randomly selected participants were included and followed on a voluntary basis are thus not representative of the general population. In addition, data were not weighted as it is usually done in large cohorts such as the National Health and Nutrition Examination Survey (NHANES) while aiming at examining the relations between several variables rather than computing representative prevalence [55, 56]. In particular, most of the participants had a favorable social context, and they are more interested in their health. Thus, our results should be extrapolated with caution to other settings. Second, when dealing with substance use, participants tend to underestimate their consumption in relation with social desirability; hence, there is a risk of an under-estimation which is a common method bias [57]. Third, even if we had a large set of sociodemographic and clinical factors for considering potential confounding effects in order to examine longitudinal associations, we cannot exclude the possibility of residual confounding due to unmeasured factors such as personality traits, or other exposure and socioeconomical factors. Thus, our findings, although computed from prospective analyses, must not be interpreted as causality pathways. Fourth, the absence of consumed quantities of fat and sugar intakes limited our ability to calculate energy intakes from these macronutrients and quantify their association with the indicators of atypical working hours. Fifth, we had one single measure of the exposures thus limiting our capacity to examine changes in the exposures before the assessment of the outcomes. Nevertheless, we examined changes in the associations according to the duration of the exposures for whom this information was available (i.e., night shifts and weekend work) and results showed that associations were more pronounced in individuals who were exposed for less than 1 year compared to those who were exposed for a longer period.” Sixth, in this present study, information on hours of sleep were not examined although this variable can be important to be taken into consideration while dealing with the associations between atypical working hours, substance use and diet rich in sugar and fat. Thus, future studies should plan to consider this variable in their models.

Working at night was associated with increased tobacco use in women, with increased cannabis use in men and with increased alcohol use in both men and women. Regarding alcohol use, this finding was not consistent with a previous published study that showed that working at night was associated with a decreased risk of being at risk [58]. Working at night could be associated with sleep disorders and fatigue [59]. Hence, workers who work at night might use these substances as sleep aids/hypnotic substances for sleep disorders or psychostimulants to overcome fatigue [60]. They might also use them to alleviate stress including psychological and work stress [61] since these individuals are more exposed to lack of peer support, as well as social and family conflicts (i.e., inter-marital tensions, imbalance in parenthood and household activities) [60]. Weekend work was associated with increased tobacco use in women and alcohol use in both men and women. Workers obliged to work on weekends have an increased likelihood of work-family conflicts compared to other workers [62]. Work-family conflicts are known to be associated with tobacco and alcohol use [63, 64]. Non-fixed working hours were associated with increased tobacco and alcohol use in both men and women. At least for some workers, non-fixed hours could increase time management autonomy (i.e., managing schedules and deadlines with minimum supervision). This situation has been associated with an increased level of work stress which could be associated with tobacco and alcohol use [65]. Moreover, since substance use are commonly associated with a decreased likelihood of finding a job [66], substance users might be oversampled in jobs that are usually less appealing, such as those requiring to work at night, on weekends or on non-fixed hours.

Stratified analyses suggested that the associations between atypical working hours and substance use may concern mainly workers from low occupational grade. This finding was consistent with the well-known increased vulnerability to substance use in workers from lower social positions [67]. Stratified analyses also suggested that the associations between atypical working hours and substance use may already appear among recently exposed workers. Although a healthy worker effect could be involved, these results could indicate that that it is probably not necessary to be exposed for a long time to observe a significant association with substance use.

Lastly, working after midnight and on Saturdays were associated with decreased sugar and fat consumption in both men and women. These work conditions might be associated with time-related barriers to eat and/or to buy food. As some atypical working hours such as night shifts are associated with a higher BMI level, the associations with atypical working hours and fat and sugar dietary patterns may be more pronounced among overweight or obese individuals or individuals following a current diet or individuals with a sedentary lifestyle. However, in the present study we failed to find significant interactions between BMI, physical activity, current diet and atypical working hours and in particular with working after midnight and on Saturdays. Moreover, we have analyzed the association between atypical working hours and fat and sugar intake by adding interaction terms in separate models with BMI, physical activity and following a particular diet in order to further explore whether our associations could be moderated by a third factor. However, interactions were not significant so it is unlikely in this study that different categories of BMI, physical activity or following or not a diet may have substantial roles in the associations between atypical working hours and fat and sugar intake. Otherwise, workers with healthier habits, including low sugar and fat intakes, might be more prone to engage themselves in jobs having high demand, such as those requiring to work after midnight or on Saturdays.

## Conclusions

To conclude, these findings should be considered in health promotion programs and prevention strategies regarding poor health outcomes associated with atypical working hours. For workers who experience atypical hours, regular monitoring with standardized screening and early intervention on substance use are needed, even among those who are recently exposed and exposed to just one type of atypical hours. Our findings suggest that workers from low occupational grade may be more concerned. Longitudinal studies with more repeated measures should examine whether subtracting the workers exposed to working atypical working hours could decrease their risk of substance use. Furthermore, qualitative studies may be important to better understand the mechanisms that underlie these associations and the sex differences.

## Supplementary Information

**Additional file 1: Supplementary Table S1.** The distribution of employees by periods of follow-up.

**Additional file 2: Supplementary Table S2.** The principal component analysis of the qualitative food frequency questionnaire using the Varimax rotation.

**Additional file 3: Supplementary Table S3.** Baseline characteristics of the employees by indicators of atypical working hours in men between 2012-2017.

**Additional file 4: Supplementary Table S4.** Baseline characteristics of the employees by indicators of atypical working hours in women between 2012-2017.

**Additional file 5: Supplementary Table S5.** Baseline characteristics of the employees by indicators of atypical working hours in men between 2012-2016.

**Additional file 6: Supplementary Table S6.** Baseline characteristics of the employees by indicators of atypical working hours in women between 2012-2017.

## Data Availability

Personal health data underlying the findings of our study are not publicly available due to legal reasons related to data privacy protection. However, the data are available upon request to all interested researchers after authorization of the French “Commission nationale de l’informatique et des libertés”. The CONSTANCES email address is contact@constances.fr. GA declares personal fees from Pierre Fabre, Lundbeck, Zentiva and Pfizer, outside the submitted work. AD declares personal fees from his mentioned affiliations, Elsevier Masson, outside the submitted work. CL declares personal fees from Boehringer Ingelheim, Janssen-Cilag, Lundbeck and Otsuka Pharmaceutical, outside the submitted work.
