# Supplementary material for: Atypical working hours are associated with tobacco, cannabis and alcohol use: longitudinal analyses from the CONSTANCES cohort
Source: BMC Public Health. 2022 Sep 29;22:1834. doi: 10.1186/s12889-022-14246-x (PMC9523930; doi:10.1186/s12889-022-14246-x)
Supplement: Supplementary file 1 — Additional file 1: Supplementary Table S1. The distribution of employees by periods of follow-up. [file 12889_2022_14246_MOESM1_ESM.docx]

**Additional file 1**

**Supplementary Table S1.** **The distribution of employees by periods of follow-up.**

| Study outcomes | Periods of follow-up | | | | | |
| --- | --- | --- | --- | --- | --- | --- |
|  | 2012-2013 | 2013-2014 | 2014-2015 | 2015-2016 | 2016-2017 | 2017-2018 |
|  | N | | | | | |
| Tobacco |  | | | | | |
| Ex-smokers | 1,668 | 3,866 | 4,884 | 5,855 | 6,976 | 7,667 |
| Current smokers | 1,121 | 2,361 | 3,378 | 3,927 | 4,417 | 4,874 |
| Ever-smokers | 2,789 | 6,227 | 8,262 | 9,782 | 11,393 | 12,541 |
| Cannabis (ever-users) | 1,622 | 3,629 | 5,274 | 6,425 | 8,062 | 9,216 |
| Alcohol/diet rich in sugar and fat* | 5,283 | 12,075 | 15,858 | 19,578 | 22,620 | N/A |

*The individuals that were followed-up for diet rich in sugar and fat were followed from 2012-2016 (baseline) until 2017
