# Supplementary material for: Atypical working hours are associated with tobacco, cannabis and alcohol use: longitudinal analyses from the CONSTANCES cohort
Source: BMC Public Health. 2022 Sep 29;22:1834. doi: 10.1186/s12889-022-14246-x (PMC9523930; doi:10.1186/s12889-022-14246-x)
Supplement: Supplementary file 2 — Additional file 2: Supplementary Table S2. The principal component analysis of the qualitative food frequency questionnaire using the Varimax rotation. [file 12889_2022_14246_MOESM2_ESM.docx]

**Additional file 2**

**Supplementary Table S2. The principal component analysis of the qualitative food frequency questionnaire using the Varimax rotation.**

| **Items** | **Sugar and fat** | **Traditional diet** | **Low fat protein** |
| --- | --- | --- | --- |
| How much sugar (white, brown etc.) do you consume per day (in coffee, tea, yoghurt etc.)? (number of cubes or spoons) | 0.29 | -0.16 | -0.08 |
| How much sweetener (aspartame, stevia, agave syrup etc.) do you consume per day (in coffee, tea, yoghurt etc.)? (number of cubes or spoons) | -0.05 | 0.03 | 0.19 |
| Do you enjoy very salty food, or do you add salt to your food before having tasted it? | -0.11 | 0.17 | -0.05 |
| Usually, how often do you eat meat (beef, veal, lamb, pork, etc.)? | **0.47** | 0.10 | -0.21 |
| Usually, how often do you eat poultry (chicken, turkey, etc.)? | 0.32 | 0.20 | 0.02 |
| Usually, how often do you eat fish or seafood? | -0.05 | **0.49** | 0.03 |
| Usually, how often do you eat eggs? | 0.07 | **0.40** | 0.04 |
| Usually, how often do you eat delicatessen and offal (ham, pâté, bacon, black pudding, chitterling sausage, etc.)? | **0.52** | 0.07 | -0.14 |
| Usually, how often do you drink milk? | 0.22 | 0.09 | 0.03 |
| Usually, how often do you eat dairy products (milk, petit Suisse cheese, yoghurt, cottage cheese, etc.)? | 0.21 | 0.32 | -0.14 |
| Usually, how often do you eat sweet desserts (puddings, creamy desserts, fromage frais, etc.)? | **0.56** | 0.05 | 0.11 |
| Usually, how often do you eat dairy products and desserts “low-fat” (0% or 20%? | -0.01 | 0.17 | **0.43** |
| Usually, how often do you eat cheeses “low-fat”? | 0.01 | 0.13 | **0.50** |
| Usually, how often do you eat cheeses? | 0.20 | 0.37 | -0.28 |
| Usually, how often do you eat white bread, breakfast rusks? | 0.40 | 0.04 | -0.36 |
| Usually, how often do you eat whole wheat bread, buckwheat bread, whole grain bread, rye bread, whole wheat breakfast rusks? | -0.27 | **0.46** | 0.26 |
| Usually, how often do you eat starchy foods (pasta, potatoes, rice, semolina, etc.)? | 0.37 | 0.30 | -0.11 |
| Usually, how often do you eat breakfast cereal? | 0.00 | 0.13 | 0.25 |
| Usually, how often do you eat brown rice, whole wheat pasta, etc.? | -0.19 | 0.29 | **0.44** |
| Usually, how often do you eat dried legumes (lentils, white kidney beans, broad beans, chick peas, etc.)? | -0.09 | **0.40** | 0.26 |
| Usually, how often do you eat raw or cooked vegetables? | -0.19 | **0.64** | -0.17 |
| Usually, how often do you eat fresh fruit (including squeezed)? | -0.21 | **0.63** | -0.12 |
| Usually, how often do you eat shop-bought ready meals (tinned, frozen, delicatessen: couscous, sausage and bean hotpot, sauerkraut, etc.)? | **0.48** | -0.07 | 0.29 |
| Usually, how often do you eat shop-bought ready meals “low-fat’? | 0.15 | -0.02 | **0.52** |
| Usually, how often do you eat fast food meals (hamburgers, kebabs, sandwiches, pizzas, quiches, etc.)? | **0.57** | -0.15 | 0.22 |
| Usually, how often do you eat fried food (chips, crisps, doughnuts, battered meat or fish, etc.)? | **0.64** | -0.10 | 0.16 |
| Usually, how often do you eat crisps, crackers, peanuts and other snacks? | **0.50** | 0.04 | 0.05 |
| Usually, how often do you eat pastries, cakes, vienoiseries? | **0.54** | 0.13 | 0.10 |
| Usually, how often do you eat sweet biscuits, chocolate bars or cereal bars, sweets, chocolate, etc.? | 0.39 | 0.23 | -0.03 |
| Usually, how often do you eat sweet biscuits, chocolate bars or cereal bars, sweets, chocolate, etc. “low-fat”? | 0.14 | 0.03 | **0.47** |
| Usually, how often do you eat butter, margarine (at breakfast, on the side, when preparing meals) | 0.20 | 0.34 | -0.35 |
| Usually, how often do you eat oil (seasoning or cooking)? | -0.01 | 0.05 | -0.01 |

Bold characters show the items extracted for each factor
