# Supplementary material for: Atypical working hours are associated with tobacco, cannabis and alcohol use: longitudinal analyses from the CONSTANCES cohort
Source: BMC Public Health. 2022 Sep 29;22:1834. doi: 10.1186/s12889-022-14246-x (PMC9523930; doi:10.1186/s12889-022-14246-x)
Supplement: Supplementary file 3 — Additional file 3: Supplementary Table S3. Baseline characteristics of the employees by indicators of atypical working hours in men between 2012-2017. [file 12889_2022_14246_MOESM3_ESM.docx]

**Additional file 3**

**Supplementary Table S3. Baseline characteristics of the employees by indicators of atypical working hours in men between 2012-2017.**

|  | Do you have (or have you had) work and travel times requiring you not to sleep at night at least 50 days/year? | Do you have (or have you had) work and travel times requiring you to go to bed after midnight at least 50 days/year? | Do you have (or have you had) more than one in two Sundays during the year? | Do you have (or have you had) more than one in two Saturdays during the year? | Do you work the same number of hours each day? | Do you work the same number of days each week? | Do you work fixed hours? |
| --- | --- | --- | --- | --- | --- | --- | --- |
|  | N=4,585 | N=7,144 | N=6,229 | N=12,072 | N=22,468 | N=10,989 | N=20,095 |
| Mean (SD) age, years | 44.5(10.2) | 43.2(10.9) | 43.8(11.1) | 44.0(11.3) | 44.3(10.8) | 43.9(10.8) | 44.1(10.7) |
| *P* | **<0.0001** | **<0.0001** | 0.90 | **0.0002** | **<0.0001** | **0.046** | **<0.0001** |
| Occupational grade, % |  |  |  |  |  |  |  |
| Low | 54.0 | 46.9 | 53.1 | 53.2 | 29.7 | 40.6 | 28.9 |
| Medium | 30.8 | 26.2 | 26.8 | 23.8 | 25.7 | 28.2 | 20.5 |
| High | 15.2 | 26.9 | 20.1 | 23.0 | 44.6 | 31.2 | 50.6 |
| *P* | **<0.0001** | **<0.0001** | **<0.0001** | **<0.0001** | **<0.0001** | **<0.0001** | **<0.0001** |
| Educational level using the 2011 ISCED, % |  |  |  |  |  |  |  |
| Levels 0 to 1 | 4.8 | 4.0 | 4.3 | 4.6 | 2.4 | 3.0 | 2.4 |
| Level 2 | 6.6 | 5.2 | 5.7 | 5.5 | 2.9 | 4.2 | 3.0 |
| Levels 3 to 4 | 52.4 | 42.7 | 46.6 | 43.9 | 27.8 | 38.1 | 27.2 |
| Levels 5 to 6 | 26.6 | 28.2 | 27.5 | 28.9 | 34.1 | 31.5 | 31.3 |
| Levels 7 to 8 | 9.6 | 19.9 | 15.9 | 17.2 | 32.8 | 23.2 | 36.1 |
| *P* | **<0.0001** | **<0.0001** | **<0.0001** | **<0.0001** | **<0.0001** | **<0.0001** | **<0.0001** |
| Household income in euros per month, % |  |  |  |  |  |  |  |
| <2100 | 23.4 | 22.9 | 27.1 | 26.5 | 16.7 | 22.4 | 15.2 |
| 2100-2800 | 19.8 | 18.0 | 19.2 | 18.7 | 13.7 | 15.4 | 12.8 |
| 2800-4200 | 36.6 | 33.2 | 32.2 | 32.2 | 32.4 | 33.2 | 31.0 |
| >4200 | 20.2 | 25.9 | 21.5 | 22.5 | 37.2 | 29.0 | 41.0 |
| *P* | **<0.0001** | **<0.0001** | **<0.0001** | **<0.0001** | **<0.0001** | **<0.0001** | **<0.0001** |
| Depression*, % | 11.1 | 11.0 | 10.9 | 11.0 | 9.8 | 10.8 | 9.6 |
| *P* | **0.002** | **<0.0001** | **0.0009** | **<0.0001** | 0.27 | **<0.0001** | 0.70 |

*Depression was assessed at baseline using the presence of a treated depression.

ISCED: International Standard Classification of Education.

Independent t-tests and Chi-square tests were computed for continuous and categorical variables, respectively.
