# Supplementary material for: Atypical working hours are associated with tobacco, cannabis and alcohol use: longitudinal analyses from the CONSTANCES cohort
Source: BMC Public Health. 2022 Sep 29;22:1834. doi: 10.1186/s12889-022-14246-x (PMC9523930; doi:10.1186/s12889-022-14246-x)
Supplement: Supplementary file 4 — Additional file 4: Supplementary Table S4. Baseline characteristics of the employees by indicators of atypical working hours in women between 2012-2017. [file 12889_2022_14246_MOESM4_ESM.docx]

**Additional file 4**

**Supplementary Table S4. Baseline characteristics of the employees by indicators of atypical working hours in women between 2012-2017.**

|  | Do you have (or have you had) work and travel times requiring you not to sleep at night at least 50 days/year? | Do you have (or have you had) work and travel times requiring you to go to bed after midnight at least 50 days/year? | Do you have (or have you had) more than one in two Sundays during the year? | Do you have (or have you had) more than one in two Saturdays during the year? | Do you work the same number of hours each day? | Do you work the same number of days each week? | Do you work fixed hours? |
| --- | --- | --- | --- | --- | --- | --- | --- |
|  | N=2,928 | N=4,453 | N=7,734 | N=15,136 | N=26,489 | N=13,005 | N=19,237 |
| Mean (SD) age, years | 42.7(11.0) | 41.3(11.2) | 42.4(11.4) | 42.7(11.5) | 43.6(10.8) | 43.6(10.7) | 43.5(10.7) |
| *P* | **0.002** | **<0.0001** | **<0.0001** | **<0.0001** | **<0.0001** | **<0.0001** | **<0.0001** |
| Occupational grade, % |  |  |  |  |  |  |  |
| Low | 35.7 | 41.2 | 52.6 | 55.8 | 35.6 | 40.5 | 35.0 |
| Medium | 49.1 | 34.5 | 34.7 | 29.6 | 34.3 | 37.5 | 28.4 |
| High | 15.2 | 24.3 | 12.7 | 14.6 | 30.1 | 22.0 | 36.6 |
| *P* | **<0.0001** | **0.002** | **<0.0001** | **<0.0001** | **<0.0001** | **<0.0001** | **<0.0001** |
| Educational level using the 2011 ISCED, % |  |  |  |  |  |  |  |
| Levels 0 to 1 | 1.7 | 1.9 | 3.1 | 3.1 | 1.5 | 1.8 | 1.5 |
| Level 2 | 3.9 | 4.1 | 5.6 | 5.5 | 3.2 | 3.7 | 3.3 |
| Levels 3 to 4 | 31.5 | 28.5 | 38.2 | 38.1 | 23.9 | 30.6 | 25.3 |
| Levels 5 to 6 | 48.3 | 41.7 | 39.3 | 38.2 | 43.2 | 42.5 | 39.2 |
| Levels 7 to 8 | 14.6 | 23.8 | 13.8 | 15.0 | 28.8 | 21.4 | 30.7 |
| *P* | **<0.0001** | 0.17 | **<0.0001** | **<0.0001** | **<0.0001** | **<0.0001** | **<0.0001** |
| Household income in euros per month, % |  |  |  |  |  |  |  |
| <2100 | 24.6 | 29.2 | 32.0 | 30.1 | 21.7 | 26.3 | 21.1 |
| 2100-2800 | 17.1 | 18.0 | 20.4 | 20.8 | 16.0 | 16.7 | 15.6 |
| 2800-4200 | 35.5 | 30.3 | 31.0 | 30.9 | 31.4 | 33.1 | 30.4 |
| >4200 | 22.8 | 22.5 | 16.6 | 19.2 | 30.9 | 23.9 | 32.9 |
| *P* | **<0.0001** | **<0.0001** | **<0.0001** | **<0.0001** | **<0.0001** | **<0.0001** | **<0.0001** |
| Depression*, % | 18.5 | 18.6 | 18.6 | 18.8 | 18.1 | 17.8 | 17.7 |
| *P* | 0.42 | 0.15 | **0.04** | **<0.0001** | 0.09 | 0.66 | 0.55 |

*Depression was assessed at baseline using the presence of a treated depression.

ISCED: International Standard Classification of Education.

Independent t-tests and Chi-square tests were computed for continuous and categorical variables, respectively.
