# Supplementary material for: Atypical working hours are associated with tobacco, cannabis and alcohol use: longitudinal analyses from the CONSTANCES cohort
Source: BMC Public Health. 2022 Sep 29;22:1834. doi: 10.1186/s12889-022-14246-x (PMC9523930; doi:10.1186/s12889-022-14246-x)
Supplement: Supplementary file 5 — Additional file 5: Supplementary Table S5. Baseline characteristics of the employees by indicators of atypical working hours in men between 2012-2016. [file 12889_2022_14246_MOESM5_ESM.docx]

**Additional file 5**

**Supplementary Table S5. Baseline characteristics of the employees by indicators of atypical working hours in men between 2012-2016.**

|  | Do you have (or have you had) work and travel times requiring you not to sleep at night at least 50 days/year? | Do you have (or have you had) work and travel times requiring you to go to bed after midnight at least 50 days/year? | Do you have (or have you had) more than one in two Sundays during the year? | Do you have (or have you had) more than one in two Saturdays during the year? | Do you work the same number of hours each day? | Do you work the same number of days each week? | Do you work fixed hours? |
| --- | --- | --- | --- | --- | --- | --- | --- |
|  | N=3,462 | N=5,428 | N=4,704 | N=9,087 | N=16,728 | N=8,275 | N=14,988 |
| Mean (SD) age, years | 44.7(10.2) | 43.3(10.9) | 44.0(11.2) | 44.5(11.2) | 43.7(11.02) | 43.8(11.2) | 43.8(11.1) |
| *P* | **<0.0001** | **<0.0001** | 0.45 | 0.07 | **<0.0001** | **0.005** | **<0.0001** |
| Occupational grade, % |  |  |  |  |  |  |  |
| Low | 52.5 | 45.9 | 52.1 | 52.1 | 28.9 | 39.3 | 28.3 |
| Medium | 31.9 | 26.5 | 27.3 | 24.3 | 25.8 | 28.8 | 20.3 |
| High | 15.6 | 27.6 | 20.6 | 23.6 | 45.3 | 31.9 | 51.4 |
| *P* | **<0.0001** | **<0.0001** | **<0.0001** | **<0.0001** | **<0.0001** | **<0.0001** | **<0.0001** |
| Educational level using the 2011 ISCED, % |  |  |  |  |  |  |  |
| Levels 0 to 1 | 4.7 | 4.2 | 4.3 | 4.4 | 2.4 | 2.9 | 2.3 |
| Level 2 | 6.5 | 5.3 | 6.0 | 5.8 | 3.0 | 4.2 | 3.1 |
| Levels 3 to 4 | 52.6 | 42.5 | 46.9 | 44.2 | 27.8 | 38.2 | 27.3 |
| Levels 5 to 6 | 26.9 | 28.1 | 27.3 | 28.6 | 34.2 | 31.4 | 31.3 |
| Levels 7 to 8 | 9.3 | 19.9 | 15.5 | 17.0 | 32.6 | 23.3 | 36.0 |
| *P* | **<0.0001** | **<0.0001** | **<0.0001** | **<0.0001** | **<0.0001** | **<0.0001** | **<0.0001** |
| Household income in euros per month, % |  |  |  |  |  |  |  |
| <2100 | 23.6 | 23.3 | 27.3 | 26.4 | 16.4 | 22.2 | 15.1 |
| 2100-2800 | 19.8 | 17.9 | 19.1 | 18.8 | 14.0 | 15.7 | 12.8 |
| 2800-4200 | 35.8 | 32.4 | 32.1 | 32.1 | 31.9 | 32.7 | 30.4 |
| >4200 | 20.8 | 26.4 | 21.5 | 22.7 | 37.7 | 29.4 | 41.7 |
| *P* | **<0.0001** | **<0.0001** | **<0.0001** | **<0.0001** | **<0.0001** | **<0.0001** | **<0.0001** |
| Depression*, % | 11.2 | 11.1 | 10.8 | 10.9 | 9.9 | 10.9 | 9.7 |
| *P* | **0.02** | **0.0003** | **0.03** | **0.0003** | 0.66 | **<0.0001** | 0.34 |

*Depression was assessed at baseline using the presence of a treated depression.

ISCED: International Standard Classification of Education.

Independent t-tests and Chi-square tests were computed for continuous and categorical variables, respectively.
