# Supplementary material for: Atypical working hours are associated with tobacco, cannabis and alcohol use: longitudinal analyses from the CONSTANCES cohort
Source: BMC Public Health. 2022 Sep 29;22:1834. doi: 10.1186/s12889-022-14246-x (PMC9523930; doi:10.1186/s12889-022-14246-x)
Supplement: Supplementary file 6 — Additional file 6: Supplementary Table S6. Baseline characteristics of the employees by indicators of atypical working hours in women between 2012-2017. [file 12889_2022_14246_MOESM6_ESM.docx]

**Additional file 6**

**Supplementary Table S6. Baseline characteristics of the employees by indicators of atypical working hours in women between 2012-2017.**

|  | Do you have (or have you had) work and travel times requiring you not to sleep at night at least 50 days/year? | Do you have (or have you had) work and travel times requiring you to go to bed after midnight at least 50 days/year? | Do you have (or have you had) more than one in two Sundays during the year? | Do you have (or have you had) more than one in two Saturdays during the year? | Do you work the same number of hours each day? | Do you work the same number of days each week? | Do you work fixed hours? |
| --- | --- | --- | --- | --- | --- | --- | --- |
|  | N=2,150 | N=3,308 | N=5,728 | N=11,245 | N=19,572 | N=9,545 | N=14,221 |
| Mean (SD) age, years | 43.1(10.9) | 41.5(11.5) | 42.6(11.5) | 43.0(11.5) | 43.2(11.1) | 42.7(11.4) | 43.2(11.3) |
| *P* | 0.07 | **<0.0001** | **<0.0001** | **<0.0001** | **<0.0001** | **<0.0001** | **<0.0001** |
| Occupational grade, % |  |  |  |  |  |  |  |
| Low | 36.0 | 41.1 | 52.3 | 55.3 | 35.2 | 40.2 | 34.6 |
| Medium | 48.5 | 33.9 | 34.7 | 29.9 | 34.3 | 37.3 | 28.3 |
| High | 15.5 | 25.0 | 13.0 | 14.8 | 30.5 | 22.5 | 37.1 |
| *P* | **<0.0001** | 0.34 | **<0.0001** | **<0.0001** | **<0.0001** | **<0.0001** | **<0.0001** |
| Educational level using the 2011 ISCED, % |  |  |  |  |  |  |  |
| Levels 0 to 1 | 1.7 | 2.0 | 3.1 | 3.2 | 1.5 | 1.8 | 1.5 |
| Level 2 | 3.9 | 4.3 | 5.7 | 5.9 | 3.4 | 4.0 | 3.5 |
| Levels 3 to 4 | 32.6 | 28.6 | 39.0 | 38.4 | 24.2 | 30.8 | 25.8 |
| Levels 5 to 6 | 47.5 | 41.2 | 38.6 | 37.6 | 43.2 | 42.0 | 38.9 |
| Levels 7 to 8 | 14.3 | 23.9 | 13.6 | 14.9 | 27.7 | 21.4 | 30.3 |
| *P* | **<0.0001** | 0.22 | **<0.0001** | **<0.0001** | **<0.0001** | **<0.0001** | **<0.0001** |
| Household income in euros per month, % |  |  |  |  |  |  |  |
| <2100 | 25.2 | 29.6 | 33.1 | 30.9 | 21.9 | 26.8 | 21.5 |
| 2100-2800 | 16.7 | 17.9 | 20.1 | 20.4 | 16.0 | 16.7 | 15.5 |
| 2800-4200 | 35.8 | 29.9 | 30.6 | 30.6 | 31.2 | 32.8 | 30.0 |
| >4200 | 22.3 | 22.6 | 16.2 | 18.1 | 30.9 | 23.7 | 33.0 |
| *P* | **<0.0001** | **<0.0001** | **<0.0001** | **<0.0001** | **<0.0001** | **<0.0001** | **<0.0001** |
| Depression*, % | 18.9 | 18.8 | 18.8 | 19.1 | 18.2 | 18.0 | 17.8 |
| *P* | 0.51 | 0.43 | 0.17 | **0.0009** | 0.76 | 0.97 | 0.26 |

*Depression was assessed at baseline using the presence of a treated depression.

ISCED: International Standard Classification of Education.

Independent t-tests and Chi-square tests were computed for continuous and categorical variables, respectively.
